# Intelligence, Correspondence, &c.

**Published:** 1832-10-01

**Authors:** 


### INTELLIGENCE, CORRESPONDENCE, &c.

We are glad to find that Dr. Carswell is preparing for publication a work on Pathology, illustrated by coloured representations of diseases. From the long period of time during which Dr. Carswell has devoted his attention to pathology, and his power of delineating disease, we anticipate a work which is much wanted, and which, we have no doubt, will do credit to himself, and be of benefit to the profession at large.

We have to acknowledge the receipt of a phial (from Atherstone) containing some animals evacuated from the bowels of a man, and supposed to have been generated there, after drinking out of a trough of water in Derbyshire. One of them measures 2½ inches and has 152 legs. We refer the gentleman who favoured us with these animals to a paper by Dr. Pockells in our Journal a few years back, on the same subject.

